# Two new endophytic *Colletotrichum* species from *Nothapodytespittosporoides* in China

**DOI:** 10.3897/mycokeys.49.31904

**Published:** 2019-03-11

**Authors:** Sixuan Zhou, Lijun Qiao, Ruvishika S. Jayawardena, Kevin D. Hyde, Xiaoya Ma, Tingchi Wen, Jichuan Kang

**Affiliations:** 1 Engineering Research Center of the Utilization for Characteristic Bio-Pharmaceutical Resources in Southwest, Ministry of Education/College of Life Sciences, Guizhou University, Guiyang, Guizhou Province 550025, China Guizhou University Guiyang China; 2 Institute of Animal Husbandry and Veterinary, Guizhou Academy of Agricultural Sciences, Guiyang, Guizhou province 550006, China Guizhou Academy of Agricultural Sciences Guiyang China; 3 Center of Excellence in Fungal Research, Mae Fah Luang University, Chiang Rai 57100, Thailand Mae Fah Luang University Chiang Rai Thailand

**Keywords:** Ascomycota, Multi-loci, Phylogeny, Morphology, Taxonomy

## Abstract

Two new endophytic species, *Colletotrichumjishouense***sp. nov.** and. *C.tongrenense***sp. nov.** were isolated from *Nothapodytespittosporoides* in Guizhou and Hunan provinces, China. Detailed descriptions and illustrations of these new taxa are provided and morphological comparisons with similar taxa are explored. Phylogenetic analysis with combined sequence data (ITS, GAPDH, ACT and TUB2) demonstrated that both species formed distinct clades in this genus. This is the first record of *Colletotrichum* species from *N.pittosporoides* in China.

## Introduction

*Nothapodytespittosporoides* (Oliv.) Sleum (Icacinacceae) has been used as Traditional Chinese Medicine (TCM) and is mainly distributed in southern China (Fang 1981). It is quickly gaining attention as the characteristic compounds of camptothecin and its derivatives (CIDs) in *N.pittosporoides* (Dong et al. 2015) are used as anti-cancer drugs in the world market (Demain and Vaishnav 2011). It is recognised that endophytes reside in the internal tissues of living plants and potentially have the capability to produce the same functional compounds as their hosts (Stierle et al. 1993, 1995; Kusari et al. 2008; Bhalkar et al. 2016; Uzma et al. 2018). The endophytic fungi in *N.pittosporoides* were therefore studied for their secondary metabolites with pharmaceutical potential.

Endophytic fungi were isolated from different parts of *Nothapodytespittosporoides* (Zhou et al. 2017; Qiao et al. 2018) collected from different sites. A high diversity of fungi were found, of which several species of *Colletotrichum* were isolated and identified.

*Colletotrichum* species are globally distributed and occur in various plants as endophytes (Tibpromma et al. 2018). *Colletotrichum* is the sole genus in the family Glomerellaceae (Glomerellales, Sordariomycetes, Wijayawardene et al. 2018) and was introduced by Corda (1831) with the type species *C.lineola* (Jayawardena et al. 2016, 2017, Wijayawardene et al. 2017). Recently, several studies have analysed this genus and these are summarised in Hyde et al. (2014), who accepted 163 names. Since this review, about 30 more species have been introduced (Baroncelli et al. 2017; Douanla-meli et al. 2017; Jayawardena et al. 2017; Silva et al. 2018).

In this study, we introduce two novel species, *C.jishouense* sp. nov. and *C.tongrenense* sp. nov. isolated as endophytes from *N.pittosporoides*. These species are based on both morphological features and molecular sequence data evidence.

## Material and methods

### Sample collection

Fresh healthy plant samples (leaves, stems and roots) of *Nothapodytespittosporoides* were collected in Tongren City, Guizhou Province and Jishou City, Hunan Province, China. Materials were kept in zip-lock bags on ice. Fungal isolation was carried out within 24 hours of collection.

### Isolation and cultivation of fungal endophytes

Each part of the plant was surface sterilised to eliminate epiphytic microorganisms. The samples were washed thoroughly in running tap water, followed by immersion in 70% (v/v) ethanol for 3 min to sterilise the surfaces, then rinsed with sterilised distilled water for 1 min. Samples were dried on sterilised filter paper and then placed in 3% hydrogen peroxide for 7 min, washed in sterilised distilled water and dried on a sterilised filter paper again. Each plant tissue was then cut into small cubes (0.5 × 0.5 cm) using a sterilised blade. The cubes were placed on potato dextrose agar (PDA) medium in Petri dishes containing with antibiotic (100 mg/l chloramphenicol) and incubated at 25 °C until fungal growth emerged from the plant segments. The endophytic fungi were isolated and sub-cultured on fresh PDA plates at 25 °C in darkness. Fungal isolates were stored on PDA and covered with sterilised water at 4 °C.

The type specimens are deposited in Guizhou Agricultural College (GACP), Guiyang, China. Ex-type living cultures are deposited at Guizhou Medical University Culture Collection (GMBC). Mycobank numbers are provided.

### DNA extraction, PCR amplification, and sequencing

Genomic DNA was extracted from fresh fungal mycelia using the BIOMIGA Fungus Genomic DNA Extraction Kit (GD2416, Biomiga, USA), following the manufacturer’s instructions. DNA samples were stored at -20 °C until used for polymerase chain reaction (PCR). Four loci, rDNA regions of internal transcribed spacers (ITS), partial β-tubulin (TUB2), actin (ACT) and glyceraldehyde-3-phosphate dehydrogenase (GAPDH) genes were amplified by PCR with primers ITS1 (Gardes and Bruns 1993) + ITS4 (White et al. 1990), Bt-2a + Bt-2b (Glass and Donaldson 1995), ACT-512F + ACT-783R (Carbone and Kohn 1999) and GDF1 + GDR1 (Guerber et al. 2003), respectively. The components of a 50 µl volume PCR mixture were used as follows: 2.0 µl of DNA template, 1 µl of each forward and reverse primer, 25 µl of 2 × Easy *Taq*PCR Super Mix (mixture of Easy *Taq* TM DNA Polymerase, dNTPs and optimised buffer, Beijing Trans Gen Biotech Co., Chaoyang District, Beijing, China) and 19 µl sterilised water. PCR thermal cycle programmes for ITS and ACT gene amplification were provided as: initial denaturation at 95 °C for 3 min, followed by 35 cycles of denaturation at 95 °C for 30 s, annealing at 52 °C for 50 s, elongation at 72 °C for 45 s and final extension at 72 °C for 10 min. The PCR thermal cycle programme for GAPDH gene amplification was provided as: initial denaturation at 95 °C for 3 min, followed by 35 cycles of denaturation at 94 °C for 30 s, annealing at 60 °C for 30 s, elongation at 72 °C for 45 s and final extension at 72 °C for 10 min. The PCR thermal cycle programme for TUB2 gene amplification was provided as: initial denaturation 95 °C for 3 min, followed by 35 cycles of denaturation at 94 °C for 30 s, annealing at 55 °C for 45 s, elongation at 72 °C for 45 s and final extension at 72 °C for 10 min. The quality of PCR products were checked with 1.5% agarose gel electrophoresis stained with ethidium bromide. PCR products were sent for sequencing to Sangon Co., Shanghai, China.

### Sequence alignment and phylogenetic analyses

Sequence data of the four loci were blasted in the GenBank database and all top hits, including the corresponding type sequences, were retrieved (Table 1). Multiple sequence alignments for ITS, TUB2, ACT and GAPDH were constructed and carried out using the MAFFT v.7.110 online programme (http://mafft.cbrc.jp/alignment/server/, Katoh and Standley 2013) with the default settings. Four datasets of ITS, TUB2, ACT and GAPDH of *Colletotrichum* spp. were combined and manually adjusted using BioEdit v.7.0.5.3 (Hall 1999), then assembled using SequenceMatrix1.7.8 (Vaidya et al. 2011). The final alignments contained 1593 characters with gaps, ITS with 522 sites, TUB2 with 510 sites, ACT with 269 sites and GAPDH with 292 sites. Fifty-four taxa and 1593 sites were used for phylogenetic analyses. Gaps were treated as missing data in maximum likelihood (ML), Bayesian Inference (BI) and parsimony trees. The phylogeny website tools “ALTER” (Glez-Peña et al. 2010) were used to convert the alignment file from Fasta to PhyLip file for RAxML analysis and Nexus for MrBayes. All loci were tested based on single maximum likelihood (ML) trees and Bayesian Inference (BI) methods.

**Table 1.** Taxa used for phylogenetic analyses in the study.

Species name	Isolate No.^b^	GenBank Accession No.			
		ITS	GAPDH	ACT	TUB
* Colletotrichum agaves *	AR3920	DQ286221	–^a^	–	–
* C. anthrisci *	CBS 125334*	GU227845	GU228237	GU227943	GU228139
* C. aracearum *	LC1041	KX853167	KX893586	KX893578	KX893582
* C. arxii *	CBS 132511	KF687716	KF687843	KF687802	KF687881
* C. brevisporum *	BCC 38876*	JN050238	JN050227	JN050216	JN050244
* C. chlorophyte *	IMI 103806*	GU227894	GU228286	GU227992	GU228188
* C. citricola *	SXC151*	KC293576	KC293736	KC293616	KC293656
* C. citri-maximae *	AGMy0254*	KX943582	KX943578	KX943567	KX943586
* C. cliviae *	CBS 125375*	JX519223	JX546611	JX519240	JX519249
* C. coccodes *	CBS 369.75	HM171679	HM171673	HM171667	JX546873
* C. colombiense *	CBS 129818*	JQ005174	JQ005261	JQ005522	JQ005608
* C. conoides *	CAUG17*	KP890168	KP890162	KP890144	KP890174
* C. constrictum *	CBS 128504*	JQ005238	JQ005325	JQ005586	JQ005672
* C. cordylinicola *	ICMP18579*	JX010226	JX009975	HM470235	JX010440
* C. dematium *	CBS 125.25*	GU227819	GU228211	GU227917	GU228113
* C. dracaenophilum *	CBS 118199	JX519222	JX546707	JX519238	JX519247
* C. euphorbiae *	CPC 21823	KF777146	KF777131	KF777125	KF777247
* C. excelsum-altitudum *	CGMCC 3.15130*	HM751815	KC843502	KC843548	JX625211
* C. fructi *	CBS 346.37*	GU227844	GU228236	GU227942	GU228138
* C. fuscum *	CBS 133701*	KM105174	KM105524	KM105384	KM105454
* C. fusiforme *	MFLU 13-02*9*1*	KT290266	KT290255	KT290251	KT290256
* C. gigasporum *	CBS 133266	KF687715	KF687822	–	KF687866
* C. godetiae *	CBS 133.44*	JQ948402	JQ948733	JQ949723	JQ950053
* C. grevilleae *	CBS 132879*	KC297078	KC297010	KC296941	KC297102
* C. hymenocallidicola *	MFLUCC 12–0531*	KT290264	KT290263	–	–
*** C. jishouense ***	**GZU_HJ2_G2**	**MH482931**	**MH681657**	**MH708134**	**MH727472**
*** C. jishouense ***	**GZU_HJ2_G3**	**MH482929**	**MH681658**	**MH708135**	**MH727473**
*** C. jishouense ***	**GZU_HJ2_G4**	**MH482932**	**MH681659**	**MH708136**	**MH727474**
*** C. jishouense ***	**GZU_HJ3_J5**	**MH482930**	**MH492706**	**MH708137**	–
* C. kahawae *	C1266.1	JX010231	JX010012	JX009452	JX010444
* C. ledebouriae *	CPC 25671*	KX228254	–	KX228357	–
* C. liaoningense *	CAUOS2*	KP890104	KP890135	KP890097	KP890111
* C. lindemuthianum *	CBS 144.31*	JQ005779	JX546712	JQ005842	JQ005863
* C. magnisporum *	CBS 398.84	KF687718	KF687842	KF687803	KF687882
* C. malvarum *	CBS 521.97*	KF178480	KF178504	KF178577	KF178601
* C. neosansevieriae *	CPC 25127*	KR476747	KR476791	KR476790	KR476797
* C. nymphaeae *	CBS 515.78	JQ948197	JQ948527	JQ949518	JQ949848
* C. orchidophilum *	CBS 632.80*	JQ948151	JQ948481	JQ949472	JQ949802
* C. pisicola *	CBS 724.97*	KM105172	KM105522	KM105382	KM105452
* C. pseudoacutatum *	CBS 436.77*	JQ948480	JQ948811	JQ949801	JQ950131
* C. pseudomajus *	CBS 571.88	KF687722	KF687826	KF687801	KF687883
* C. radices *	CBS 529.93	KF687719	KF687825	KF687785	KF687869
* C. rhombiforme *	CBS 129953*	JQ948457	JQ948788	JQ949778	JQ950108
* C. sansevieriae *	MAFF 239721*	NR_152313	–	–	–
* C. spinosum *	CBS 515.97*	KF178474	KF178498	KF178571	KF178595
* C. tanaceti *	CBS 132693*	JX218228	JX218243	JX218238	JX218233
* C. trichellum *	CBS 217.64*	GU227812	GU228204	GU227910	GU228106
*** C. tongrenense ***	**GZU_TRJ1-37**	**MH482933**	**MH705332**	**MH717074**	**MH729805**
* C. tropicicola *	L58	JN050240	JN050229	JN050218	JN050246
* C. truncatum *	CBS 151.35	GU227862	GU228254	GU227960	GU228156
* C. vietnamense *	CBS 125478	KF687721	KF687832	KF687792	KF687877
* C. yunnanense *	CBS 132135*	JX546804	JX546706	–	JX519248
* Monilochaetes infuscans *	CBS 869.96	JQ005780	JX546612	JQ005843	JQ005864

Notes: New strains are in bold. * ex-type strains. **^a^** No data in GenBank. **^b^**BCC: BIOTEC Culture Collection, National Center for Genetic Engineering and Biotechnology (BIOTEC), Khlong Luang, Pathumthani, Thailand; CBS: Culture collection of the Centraalbureau voor Schimmelcultures, Fungal Biodiversity Centre, Utrecht, The Netherlands; CGMCC: China General Microbiological Culture Collection; CPC: Working collection of Pedro W. Crous, housed at CBS; IMI: Culture collection of CABI Europe UK Centre, Egham, UK; LC: Working collection of Lei Cai, housed at CAS, China; MAFF: MAFF GenBank Project, Ministry of Agriculture, Forestry and Fisheries, Tsukuba, Japan; MFLUCC: Mae Fah Luang University Culture Collection, Chiang Rai, Thailand; MFLU: Herbarium of Mae Fah Luang University, Chiang Rai, Thailand; ICMP: International Collection of Microorganisms from Plants, Auckland, New Zealand.

**Figure 1.**
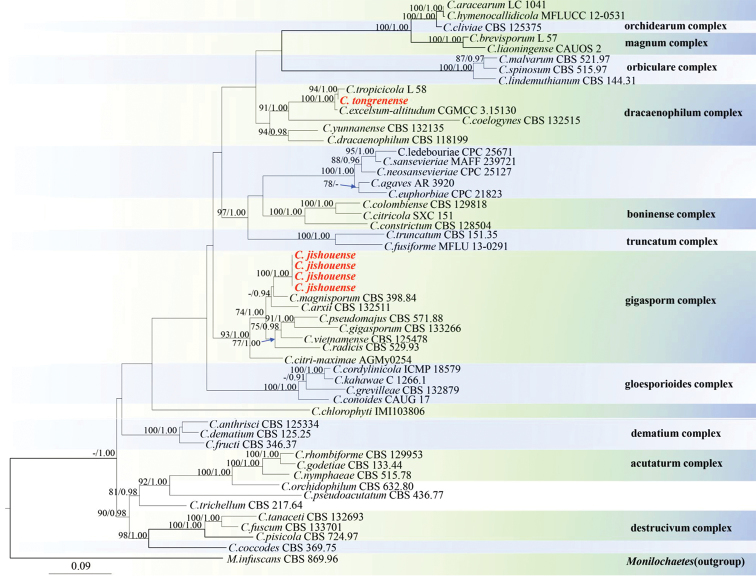
Phylogram generated from Maximum Likelihood (RAxML) analysis based on combined ITS, ACT, TUB2 and GAPDH DNA sequence data of *Colletotrichum*. Bayesian Posterior Probabilities (BSPP) greater than 0.90 and Maximum Likelihood Bootstrap Support values (MLBS) greater than 70% are shown above branches. New isolates are in red. The tree is rooted with *Monilochaetesinfuscans*CBS 869.96.

Maximum Likelihood (ML) analysis was performed on the website of CIPRES Science Gateway v.3.3 (http://www.phylo.org/portal2/, Miller et al. 2010) using RAxML-HPC Blackbox version 8.2.10. All free model parameters were estimated by RAxML and ML estimate of 25 per site rate categories. Final ML searches were conducted using the GTRGAMMA model. Bootstrap Support values (BS) equal to or greater than 60% are given above each node (Fig. 1).

For Bayesian Inference (BI), a Markov Chain Monte Carlo (MCMC) algorithm was used to generate phylogenetic trees with Bayesian probabilities using MrBayes 3.2.6 (Ronquist et al. 2012) for the combined sequence datasets. MrModeltest v.2.3 (Nylander 2004) was used to carry out the statistical selection of the best-fit model of nucleotide substitution. GTR+G model was selected for ITS, a GTR+I+G model for TUB2, a HKY+I+G model for ACT and GAPDH were incorporated into the analysis. Models of nucleotide substitution for each gene determined by MrModeltest v. 2.3 were included for each set of gene sequence data. Two runs were executed simultaneously for 1,000,000 generations and sampled every 100 generations. Of the trees, 25% were discarded as burn-in and the remaining trees were used to calculate the posterior probabilities. Convergence was assumed when the standard deviation of split sequences was less than 0.01. Phylogenetic trees were visualised using FigTree v1.4.0 (http://tree.bio.ed.ac.uk/software/figtree/, Rambaut 2012). The final alignment was deposited in Treebase (http://www.treebase.org, submission number 23622).

### Morphological analysis

Isolates were grown on PDA, water agar (WA) with bamboo and corn malt agar medium (CMA) for examination of morphological characters. Colonies were examined after 7, 14 and 21 d at 25 °C in darkness. The morphological characters of mycelia, conidiophores, conidiogenous cells and conidia were observed and photographed using a Nikon NI-SS microscope and processed with Adobe Photoshop CS3 Extended version 10.0 software (Adobe Systems, USA).

## Results

### Sample collection and isolation

Four hundred and forty endophytic fungi were isolated from different parts of *Nothapodytespittosporoides* in Jishou, Hunan Province and Tongren, Guizhou Province, belonging to twenty-four genera based on ITS sequences analysis. *Colletotrichum* was a common genus amongst the isolates. Herein, five endophytic taxa were isolated and identified as *Colletotrichum* of which GZU_HJ2_G2, GZU_HJ2_G3 and GZU_HJ2_G4 were isolated from roots and GZU_HJ3_J5 from stems of *N.pittosporoides* in Jishou, Hunan Province. GZU_TRJ1-37 was isolated from stems of *N.pittosporoides* in Tongren, Guizhou Province.

### Phylogenetic analyses

Phylogenetic analysis of four loci (ITS, GAPDH, ACT and TUB2) sequence datasets included 54 taxa, 1,593 positions including gaps (ITS: 1–522, TUB2: 523–1032, ACT: 1033–1301, GAPDH: 1302–1593) and *Monilochaetesinfuscans* (CBS 869.96) was selected as the outgroup taxon. The 50% majority rule consensus Bayesian phylogram presented in Fig. 1 and the topology is recovered with the RAxML tree. Values of the Bayesian PP ≥ 0.70 from MCMC analyses and bootstrap support values of RAxML ≥ 90% are given on the branches.

Representatives of complexes and species in *Colletotrichum* (Noireung et al. 2012; Tao et al. 2013; Liu et al. 2014; Jayawardena et al. 2016; Douanla-meli et al. 2017) are included in the phylogenetic analyses (Fig. 1). Four isolates, GZU_HJ2_G2, GZU_HJ2_G3, GZU_HJ2_G4 and GZU_HJ3_J5, were identified as distinct new species and are described as *Colletotrichumjishouense* sp. nov., and as *C.tongrenense* sp. nov., based on their morphology and molecular phylogeny.

### Taxonomy

#### Colletotrichum jishouense

Taxon classificationFungiGlomerellalesGlomerellaceae

SX. Zhou, JC. Kang & K.D. Hyde sp. nov.

828723

Fig. 2

##### Etymology.

‘*Jishouense*’ referring to Jishou City, site of collection of type species.

##### Description.

Endophytic fungus in root of *Nothapodytespittosporoides*. ***Sexual morph***: Undetermined. ***Asexual morph***: Vegetative hyphae 0.5–1.2 µm diam. (n=10), hyaline, smooth-walled, septate, branched. *Chlamydospores* not observed. *Conidiophores* formed on a basal cushion, hyaline to pale brown, clavate or cylindrical, septate and irregularly branched. *Conidiogenous cells* 4–11 × 2–3 μm (*x*‒= 6.7 ± 3.0 × 2.6 ± 0.4 μm, n=20), L/W ratio= 2.5, hyaline, smooth-walled, clavate to mostly ampulliform or cylindrical. *Conidia* hyaline, smooth-walled, aseptate, straight, cylindrical, some clavate, the apex and base rounded, 5–14 × 3–5 μm (*x*‒ = 10.8 ± 1.8 × 3.7 ± 0.5 μm, n = 40), L/W ratio= 2.9. *Appressoria* not observed.

##### Culture characteristics.

Colonies on PDA, reaching 55–60 mm diam. in 14 days at 25 °C in darkness, circular, mycelium superficial and partially immersed, more or less planar, brown in the medium but covered with abundant, pale and lanose to cottony aerial mycelium, reverse greenish pale brown, margin entire and irregular.

##### Material examined.

CHINA, Hunan Province, Jishou City (28°55'24"N, 109°10'24"E), isolated from healthy roots of *Nothapodytespittosporoides*, 27 May 2016, S.X. Zhou (Holotype GACP GZU_HJ2_G3 dried culture), ex-type living culture, GMBC0209, living culture, GZU_HJ2_G2, living culture, GZU_HJ2_G4.

China, Hunan Province, Jishou City (28°55'24"N, 109°10'24"E), isolated from healthy stem of *Nothapodytespittosporoides*, 27 May 2016, S.X. Zhou, living culture, GZU_HJ3_J5.

##### Notes.

*Colletotrichumjishouense* belongs in the *gigasporum* species complex. *C.jishouense* has shorter and narrower conidiogenous cells and conidia than all the related species in the *C.gigasporum* complex (Liu et al. 2014). Phylogenetically, our four new isolates clustered together with *C.magnisporum* (CBS 398.84). The pairwise dissimilarities of DNA sequences between *C.jishouense* and *C.magnisporum* were 2 bp, 20 bp, 5 bp and 9 bp in ITS, TUB2, ACT and GAPDH, respectively. They are phylogenetically distinct species and, therefore, *C.jishouense* sp. nov. is introduced.

**Figure 2.**
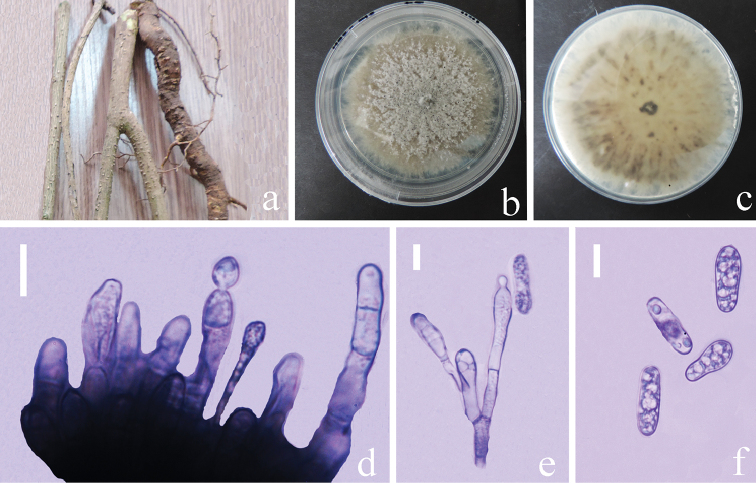
*Colletotrichumjishouense* (GACP GZU_HJ2_G3, holotype) **a** stems and roots of *Nothapodytespittosporoides***b,c** colonies on PDA**d** conidiophores in cotton blue **e** conidiophores with conidia in cotton blue **f** conidia in cotton blue. Scale bars: 10 µm (**d**), 5 µm (**e, f**).

#### Colletotrichum tongrenense

Taxon classificationFungiGlomerellalesGlomerellaceae

S.X. Zhou, J.C. Kang & K.D. Hyde sp. nov.

828725

Fig. 3

##### Etymology.

‘*tongrenense*’ referring Tongren City, site of collection of type species.

##### Description.

Endophytic in leaves and stems of *Nothapodytespittosporoides*. ***Sexual morph***: Undetermined. ***Asexual morph***: On WA, vegetative hyphae 1.4–6 µm diam. (n=10), smooth-walled, septate, branched, hyaline. *Chlamydospores* not observed. *Setae* unbranched, septate, tapering to rounded at apical end, pale brown to dark brown, smooth-walled, 45–90 µm long, 5.9–6.2 μm wide at widest part, 2.6–5.8 µm wide at bottom, 1.5–1.6 µm wide at apex. *Conidiophores* pale brown, septate, branched. *Conidiogenous cells* pale, hyaline, smooth-walled, erect, clavate or cylindrical, 2–11 × 1–2 μm (*x*‒ = 6.3 ± 4.4 × 1.7 ± 0.4 μm, n = 20), L/W ratio= 3.7. *Conidia* hyaline, aseptate, smooth-walled, variable in size and shape, thick-walled, ellipsoidal to subglobose, the apex and base rounded, slightly constricted in the middle, 11–14 × 5–7 μm (*x*‒ = 13.1 ± 1.0 × 5.5 ± 0.6 μm, n = 40), L/W ratio= 2.4.

##### Culture characteristics.

Cultures on WA at 25 °C in darkness, reaching 15–18 mm diam. in 21 days, white to grey, asymmetrical surface, reverse dark grey to black.

Colonies on PDA at 25 °C reaching 45–55 mm diam. in 12 days in darkness, circular, more or less planar, surface dark brown, covered with abundant, pale grey, lanose to cottony aerial mycelium, margin smooth, entire and pale white. Reverse dark grey, margin pale white.

Cultures on CMA, 10–15 mm diam. in 21 days, covered with dark brown aerial mycelium, sparse, reverse light brown, margin irregular.

##### Material examined.

CHINA, Guizhou province, Tongren (27°35'37"N, 109°10'58"E, elevation 332.8 m), isolated from healthy stems of *Nothapodytespittosporoides*, 27 May 2016, S.X. Zhou and L.J. Qiao (Holotype GACP GZU-TRJ1-37 dried culture), ex-type living culture, GMBC0209.

##### Notes.

*Colletotrichumtongrenense* belongs to the *C.dracaenophilum* species complex (Damm et al. 2019). Morphologically, *C.tongrenense* resembles *C.tropicicola* and *C.excelsum-altitudum* in conidia characters, but it can be distinguished from *C.tropicicola* in having setae and longer conidia (15–19 µm *vs* 11–14 µm) (Noireung et al. 2012). *C.tongrenense* is distinguished from *C.excelsum-altitudum* (Tao et al. 2013) in having smaller conidiophores (2–11 × 1–2 μm vs 8.5–25 × 4–5 μm). Phylogenetically, the new isolate GZU_TRJ1-37 clusters together with *C.tropicicola* with good bootstrap support (94% MLBS, 1.00 PP) (Fig. 1) and the phylogenetic analysis supports it as a distinct species. There are 6, 4, 2 and 5 base pairs differences in ITS, TUB2, ACT and GAPDH gene regions, respectively, between the new isolate and the type strain of *C.tropicicola*, which confirms that they are separate species. Therefore, it is introduced as a novel species.

**Figure 3.**
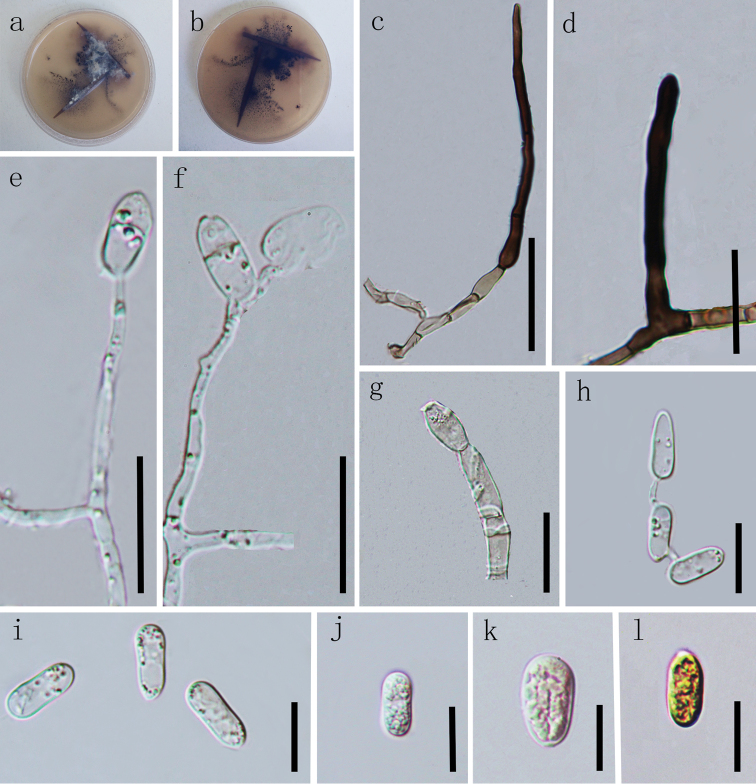
*Colletotrichumtongrenense* (GACP GZU_TRJ1-37, holotype) **a, b** colonies on WA**c–g** Conidiophores **h–l** Conidia. Scale bars: 40 µm (**c**), 20 µm (**d, g**), 10 µm (**e, f**), 10 µm (**h–l**).

## Discussion

*Colletotrichum* appears to have a wide host range and a geographic distribution (Yang et al. 2009, Hyde et al. 2014, Jayawardena et al. 2016). This study reports on five endophytic *Colletotrichum* isolates which were isolated from *Nothapodytespittosporoides*. Two new species were introduced, named *C.jishouense* and *C.tongrenense*, respectively, based on morphological characters and multilocus (ITS, TUB2, ACT and GAPDH) phylogenetic analyses. The *C.gigasporum* species complex is associated with various host plants as pathogens and endophytes and also isolated from air and stored grain, indicating that the members are not host-specific and apparently have different life styles (Than et al. 2008, Yang et al. 2009, Liu et al. 2014, Jayawardena et al. 2016). The *C.dracaenophilum* species complex contains a few apparently host-specific species and these species seem to be uncommon (Damm et al. 2019). The complex includes *C.coelogynes*, *C.dracaenophilum*, *C.excelsum-altitudinum*, *C.tropicicola* and *C.yunnanense*. A further strain, *C.tongrenense* was identified to the *C.dracaenophilum* species complex in the study, based on the multilocus phylogeny and morphological features. Amongst them, *C.excelsum-altitudinum* was described from healthy leaves of *Bletillaochracea* (Orchidaceae) in Guizhou, China (Tao et al. 2013.), *C.tropicicola* were described from leaves of *Citrusmaxima* and *Paphiopedilum* sp. in Thailand and a further strain from *C.* sp. in Mexico (Noireung et al. 2012, Damm et al. 2019). The *C.coelogynes* strain CBS 132504 is an endophytic *Colletotrichum* isolate from both *Dendrobium* spp. in China (Yuan et al. 2009, Gao and Guo, unpublished data). *C.yunnanense* was described from healthy leaves of *Buxus* sp. in Yunnan, China (Liu et al. 2007).

Morphological features and genes sequence data are recognised as a basis for describing new species, but sometimes morphological features of *Colletotrichum* are not stable and may change under different growth conditions (Liu et al. 2014). DNA sequence comparison and multi-gene phylogenetic analyses can provide sufficient evidence to show distinct taxa (Jeewon and Hyde 2016). However, single gene data, including ITS, are usually insufficient for species identification in most of the *Colletotrichum* species complexes (Hyde et al. 2009). Multi-locus phylogenies are therefore necessary to describe *Colletotrichum* species (Jayawardena et al. 2016).

The composition of endophytic microorganisms may depend on the plant age, tissue, host type and time of isolation (Rosenblueth and Martinez-Romero 2006). The new species, *Colletotrichumtongrenense* lives in stems and *C.jishouense* lives in roots and stems of *Nothapodytespittosporoides*. Nothing is known about their infection strategies on the host. It is also the first report of *Colletotrichum* species from *N.pittosporoides*. This study enriches the host diversity of *Colletotrichum*.

## Supplementary Material

XML Treatment for Colletotrichum jishouense

XML Treatment for Colletotrichum tongrenense

## References

[B1] Bhalkar BN Patil SM Govindwar SP (2016) Camptothecine production by mixed fermentation of two endophytic fungi from *Nothapodytesnimmoniana* Fungal Biology120: 873–883. 10.1016/j.funbio.2016.04.003 27268247

[B2] Baroncelli R Talhinhas P Pensec F Sukno SA Floch GL Thon MR (2017) The *Colletotrichumacutatum* species complex as a model system to study evolution and host specialization in plant pathogens. Frontiers in Microbiology 8: 2001. 10.3389/fmicb.2017.02001 PMC5641571 29075253

[B3] Carbone I Kohn LM (1999) A method for designing primer sets for speciation studies in filamentous ascomycetes. Mycologia 91: 553–556. 10.2307/3761358

[B4] Corda ACI (1831) Die Pilze Deutschlands. In: Sturm J (Ed.) Deutschlands Flora in Abbildungen nach der Natur mit Beschreibungen.Sturm, Nürnberg 3 (12), 33–64.

[B5] Damm U Sato T Alizadeh A Groenewald JZ Crous PW (2019) The *Colletotrichumdracaenophilum*, *C.magnum* and *C.orchidearum* species complexes. Studies in Mycology 92(5): 1–46. 10.1016/j.simyco.2018.04.001 29997400 PMC6030544

[B6] Demain AL Vaishnav P (2011) Natural products for cancer chemotherapy. Microbial Biotechnology 4(6): 687–699. 10.1111/j.1751-7915.2010.00221.x 21375717 PMC3815406

[B7] Douanla-meli C Unger JG (2017) Phylogenetic study of the *Colletotrichum* species on imported citrus fruits uncovers a low diversity and a new species in the *Colletotrichumgigasporum* complex. Fungal Biology 121(10): 858–868. 10.1016/j.funbio.2017.06.003 28889910

[B8] Fang WP (1981) Flora Republicae. Popularis Sinicae 46. Science Press, Beijing, Tomus, 49 pp.

[B9] Gardes M Bruns TD (1993) ITS primers with enhanced specificity for basidiomycetes – application to the identification of mycorrhizae and rusts. Molecular Ecology 2: 113–118. 10.1111/j.1365-294X.1993.tb00005.x 8180733

[B10] Glass NL Donaldson GC (1995) Development of primer sets designed for use with the PCR to amplify conserved genes from filamentous ascomycetes. Applied Environmental Microbiology 61: 1323–1330. 7747954 10.1128/aem.61.4.1323-1330.1995 PMC167388

[B11] Glezpeña D Gómezblanco D Reboirojato M Fdezriverola F Posada D (2010) ALTER: program-oriented conversion of DNA and protein alignments. Nucleic Acids Research 38: 14–18. 10.1093/nar/gkq321 PMC2896128 20439312

[B12] Guerber JC Liu B Correll JC Johnston PR (2003) Characterization of diversity in *Colletotrichumacutatum* sensu lato by sequence analysis of two gene introns, mtDNA and intron RFLPs, and mating compatibility. Mycologia 95(5): 872–895. 10.1080/15572536.2004.11833047 21148995

[B13] Guo DY Ling TJ Cai XH (2015) Chemical constituents of *Nothapodytespittosporoides* (Icacinaceae). Biochemical Systematics and Ecology 61: 293–296. 10.1016/j.bse.2015.06.039

[B14] Hall TA (1999) BioEdit: a user-friendly biological sequence alignment editor and analysis program for Windows 95/98/NT. Nucleic Acids Symposium Series 41: 95–98.

[B15] Hyde KD Cai L Cannon PF Crouch JA Crous PW Damm U Goodwin PH Chen H Johnston PR Jones EBG Liu ZY McKenzie EHC Moriwaki J Noireung P Pennycook SR Pfenning LH Prihastuti H Sato T Shivas RG Tan YP Taylor PWJ Weir BS Yang YL Zhang JZ (2009) *Colletotrichum* names in current use. Fungal Diversity 39: 147–182.

[B16] Hyde KD Nilsson RH Alias SA Ariyawansa HA Blair JE Cai L de Cock AWAM Dissanayake AJ Glockling SL Goonasekara ID Gorczak M Hahn M Jayawardena RS van Kan JAL Laurence MH Lévesque CA Li XH Liu JK Maharachchikumbura SSN Manamgoda DS Martin FN McKenzie EHC McTaggart AR Mortimer PE Nair PVR Pawłowska J Rintoul TL Shivas RG Spies CFJ Summerell BA Taylor PWJ Terhem RB Udayanga D Vaghefi N Walther G Wilk M Wrzosek M Xu JC Yan JY Zhou N (2014) One stop shop: backbones trees for important phytopathogenic genera: I. Fungal Diversity 67: 21–125. 10.1007/s13225-014-0298-1

[B17] Index Fungorum (2017) http://www.indexfungorum.org/names/Names.asp

[B18] Jayawardena RS Camporesi E Elgorban AM Bahkali AH Yan J Hyde KD (2017) A new species of *Colletotrichum* from *Sonchus* sp. in Italy. Phytotaxa 314(1): 55–63. 10.11646/phytotaxa.314.1.3

[B19] Jayawardena RS Hyde KD Damm U Cai L Liu M Li XH Zhang W Zhao WS Yan JY (2016) Notes on currently accepted species of *Colletotrichum*. Mycosphere 7: 1192–1260. 10.5943/mycosphere/si/2c/9

[B20] Jeewon R Hyde KD (2016) Establishing species boundaries and new taxa among fungi: recommendations to resolve taxonomic ambiguities. Mycosphere 7(11): 1669–1677. 10.5943/mycosphere/7/11/4

[B21] Kusari S Lamshöft M Zühlke S Spiteller M (2008) An endophytic fungus from *Hypericumperforatum* that produces hypericin. Journal of Natural Products 71: 159–16. 10.1021/np070669k 18220354

[B22] Katoh K Standley DM (2013) MAFFT multiple sequence alignment software version 7: improvements in performance and usability. Molecular Biology and Evolution 30(4): 772–780. 10.1093/molbev/mst010 23329690 PMC3603318

[B23] Liu F Cai L Crous PW Damm U (2014) The *Colletotrichumgigasporum* species complex. Persoonia 33: 83–97. 10.3767/003158514X684447 25737595 PMC4312939

[B24] Liu X Xie X Duan J (2007) *Colletotrichumyunnanense* sp. nov., a new endophytic species from *Buxus* sp. Mycotaxon 100: 137–144.

[B25] Miller MA Pfeiffer W Schwartz T (2010) Creating the CIPRES Science Gateway for inference of large phylogenetic trees. 2010 Gateway Computing Environments Workshop (GCE), IEEE, 2010. 10.1109/GCE.2010.5676129

[B26] Noireung P Phoulivong S Liu F Cai L McKenzie EHC Chukeatirote E Jones EBG Bahkali AH Hyde KD (2012) Novel species of *Colletotrichum* revealed by morphology and molecular analysis. Cryptogamie, Mycologie, 33(3): 347–362. 10.7872/crym.v33.iss3.2012.347

[B27] Nylander JAA (2004) MrModeltest v2. Program distributed by the author. Evolutionary Biology Centre, Uppsala University, Uppsala.

[B28] Qiao LJ Zhou SX Wen TC Kang JC Lei BX (2018) Diversity of endophytic fungi from *Nothapodytespittosporoides* in Guizhou Province. Mycosystema 37(1): 43–51.

[B29] Rambaut A (2012) FigTree version 1.4. http://tree.bio.ed.ac.uk/software/figtree

[B30] Ronquist F Teslenko M van der Mark P Ayres DL Darling A Höhna S Larget B Liu L Suchard MA Huelsenbeck JP (2012) MrBayes3.2: efficient Bayesian phylogenetic inference and model choice across a large model space. Systematic Biology 61(3): 539–542. 10.1093/sysbio/sys029 22357727 PMC3329765

[B31] Rosenblueth M Martinez-Romero E (2006) Bacterial endophytes and their interactions with hosts. Acta Pharmacologica Sinica 19: 827–837. 10.1094/MPMI-19-0827 16903349

[B32] Silva MD Cruz ES Veloso TGR Miranda L Pereiram OL Bocayuva MF Kasuya MCM (2018) *Colletorichumserranegrense* sp. nov., a new endophytic species from the roots of the endangered Brazilian epiphytic orchid *Cattleyajongheana*. Phytotaxa 351(2): 163–170. 10.11646/phytotaxa.351.2.4

[B33] Stamatakis A (2014) RAxML version 8: a tool for phylogenetic analysis and post-analysis of large phylogenies. Bioinformatics 30(9): 1312–1313. 10.1093/bioinformatics/btu033 24451623 PMC3998144

[B34] Stierle A Strobel G Stierle D (1993) Taxol and taxane production by *Taxomycesandreanae*, an endophytic fungus of Pacific yew. Science 260: 214–216. 10.1126/science.8097061 8097061

[B35] Stierle A Strobel G Stierle D Grothaus P Bignami G (1995) The search forataxol-producing microorganism among the endophytic fungi of the Pacific yew, *Taxusbrevifolia*. Journal of Nature Products 58: 1315–1324. 10.1021/np50123a002 7494141

[B36] Tao G Liu ZY Liu F Gao YH Cai L (2013) Endophytic *Colletotrichum* species from *Bletillaochracea* (Orchidaceae), with descriptions of seven new species. Fungal Diversity 61(1): 139–164. 10.1007/s13225-013-0254-5

[B37] Than PP Prihastuti H Phoulivong S Taylor PWJ Hyde KD (2008) *Chillianthracnose* disease caused by *Colletotrichum* species. Journal of Zhejiang University-Science B 9: 764–788. 10.1631/jzus.B0860007 18837103 PMC2565739

[B38] Tibpromma S Hyde KD Bhat JD Mortimer PE Xu JC Promputtha I Doilom M Yang JB Tang AMC Karunarathna SC (2018) Identification of endophytic fungi from leaves of Pandanaceae based on their morphotypes and DNA sequence data from southern Thailand. Mycokeys 33: 25–67. 10.3897/mycokeys.33.23670 PMC6283267 30532625

[B39] Uzma F Mohan CD Hashem A Konappa NM Rangappa S Kamath PV Singh BP Mudili V Gupta VK Siddaiah CN (2018) Endophytic fungi-alternative sources of cytotoxic compounds: a review. Frontiers in Pharmacology, 9: 309. 10.3389/fphar.2018.00309 PMC5932204 29755344

[B40] Vaidya G Lohman DJ Meier R (2011) SequenceMatrix: concatenation software for the fast assembly of multi-gene datasets with character set and codon information. Cladistics 27: 171–180. 10.1111/j.1096-0031.2010.00329.x 34875773

[B41] White TJ Bruns T Lee S Taylor JW (1990) Amplification and direct sequencing of fungal ribosomal RNA genes for phylogenetics. PCR protocols: a guide to methods and applications. Academic Press, San Diego,California 18: 315–322. 10.1016/B978-0-12-372180-8.50042-1

[B42] Wijayawardene NN Hyde KD Lumbsch T Liu JK Maharachchikumbura SSN Ekanayaka AH Tian Q Phookamsak R (2018) Outline of Ascomycota – 2017. Fungal Diversity 88: 167–263. 10.1007/s13225-018-0394-8

[B43] Wijayawardene NN Hyde KD Rajeshkumar KC Hawksworth DL Madrid H Kirk PM Braun U (2017) Notes for genera: ascomycota. Fungal Diversity 86(1): 1–594. 10.1007/s13225-017-0386-0

[B44] Wijayawardene DNN Song Y Bhat DJ McKenzie EHC Chukeatirote E Wang Y Hyde KD (2013) *Wojnowiciaviburni* sp. nov. from China and its phylogenetic placement. Sydowia 65: 181–190.

[B45] Yang YL Liu ZY Cai L Hyde KD Yu ZN McKenzie EHC (2009) *Colletotrichum*anthracnose of Amaryllidaceae. Fungal Diversity 39: 123–146.

[B46] Yuan ZL Chen YC Yang Y (2009) Diverse non-mycorrhizal fungal endophytes inhabiting an epiphytic, medicinal orchid (*Dendrobiumnobile*): estimation and characterization. World Journal of Microbiology & Biotechnology 25(2): 295–303. hhttps://doi.org/10.1007/s11274-008-9893-1

[B47] Zhou SX Qiao LJ Kang JC Hyde KD Ma XY (2017) A new species of *Monilochaetes* from *Nothapodytespittosporoides*. Phytotaxa 326(2): 129–136. 10.11646/phytotaxa.326.2.4

